# Extracellular vesicles in cancer cachexia: deciphering pathogenic roles and exploring therapeutic horizons

**DOI:** 10.1186/s12967-024-05266-9

**Published:** 2024-05-27

**Authors:** Yifeng Wang, Shengguang Ding

**Affiliations:** 1 Department of Thoracic Surgery, Affiliated Hospital 2 of Nantong University, Nantong First People’s Hospital, Nantong, 226001 P.R. China; 2 School of Medicine, Nantong University, Nantong, 226001 P.R. China

**Keywords:** Extracellular vesicles, Cancer cachexia, Muscle wasting, Adipose atrophy, Systemic inflammation, Therapeutic agents

## Abstract

Cancer cachexia (CC) is a debilitating syndrome that affects 50–80% of cancer patients, varying in incidence by cancer type and significantly diminishing their quality of life. This multifactorial syndrome is characterized by muscle and fat loss, systemic inflammation, and metabolic imbalance. Extracellular vesicles (EVs), including exosomes and microvesicles, play a crucial role in the progression of CC. These vesicles, produced by cancer cells and others within the tumor environment, facilitate intercellular communication by transferring proteins, lipids, and nucleic acids. A comprehensive review of the literature from databases such as PubMed, Scopus, and Web of Science reveals insights into the formation, release, and uptake of EVs in CC, underscoring their potential as diagnostic and prognostic biomarkers. The review also explores therapeutic strategies targeting EVs, which include modifying their release and content, utilizing them for drug delivery, genetically altering their contents, and inhibiting key cachexia pathways. Understanding the role of EVs in CC opens new avenues for diagnostic and therapeutic approaches, potentially mitigating the syndrome’s impact on patient survival and quality of life.

## Introduction

Cancer, a leading cause of mortality worldwide, is a complex disease marked by the uncontrolled proliferation and spread of abnormal cells [1]. Recent research advances have unraveled the molecular basis of tumor development, paving the way for targeted therapies and personalized medicine [2, 3]. Despite these breakthroughs, challenges persist in early detection, treatment resistance, and metastasis. A prevalent issue in advanced cancer, affecting up to 80% of patients in the terminal stage, particularly those with digestive and respiratory system cancers, is cancer-associated cachexia which directly causes around 20 to 30% of cancer deaths [4]. This complex metabolic syndrome, characterized by irreversible weight loss, muscle wasting, and systemic inflammation, arises from a mix of tumor factors, metabolic disturbances, and immune dysfunction [5]. The resulting imbalance in energy and protein metabolism leads to poorer prognosis, lower survival rates, and diminished quality of life.

Similarly, chemotherapy, a critical treatment modality in cancer care, employs anti-cancer drugs to target rapidly dividing cells [6]. These drugs include alkylating agents that damage DNA, antimetabolites that interfere with the building blocks of DNA and RNA, anti-tumor antibiotics that alter DNA, topoisomerase inhibitors that disrupt enzymes involved in DNA separation, and mitotic inhibitors that hinder structures involved in cell division [7]. Their mechanisms of action involve killing rapidly dividing cells, damaging the DNA of cancer cells, or interfering with their metabolism, effectively halting their growth and spread [7]. Recent advancements in chemotherapy have led to the development of targeted therapies that focus on specific molecules in cancer cells [8], immunotherapies that enhance the immune system’s ability to fight cancer [9], and nanotechnology-based drug delivery systems that aim to improve treatment efficacy while reducing side effects [10]. However, a significant challenge associated with chemotherapy is cachexia, a condition characterized by significant weight loss, muscle wasting, and fatigue, affecting an estimated 20–80% of patients [11, 12]. Cachexia is exacerbated by the direct impact of chemotherapeutic agents on muscle and fat metabolism, which not only diminishes the patient’s quality of life but also affects the tolerability and effectiveness of chemotherapy, increasing the risk of complications.

**Fig. 1:**
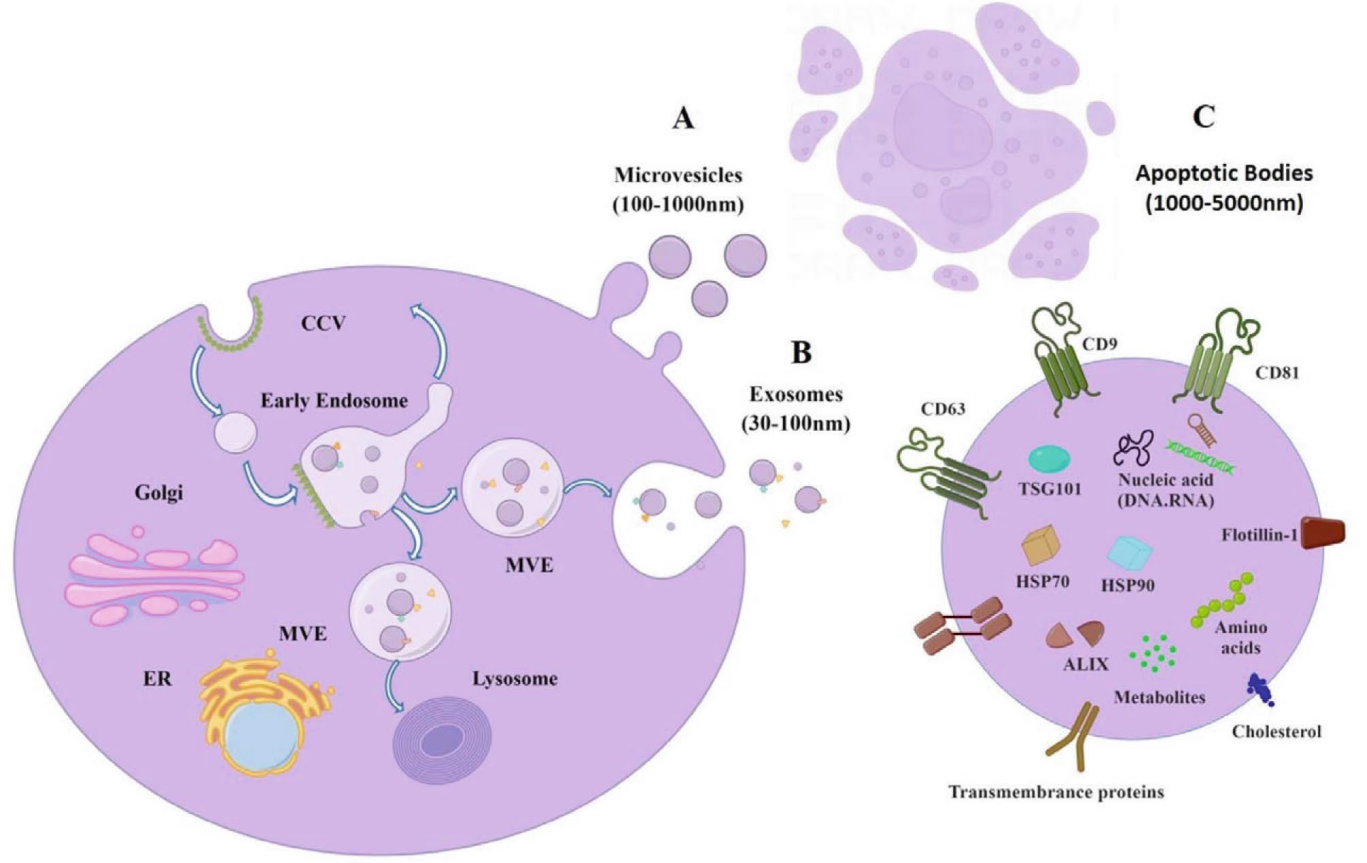
EVs: Composition and origins. EVs are small, membranous structures of varying contents, released by cells into the extracellular space. The composition and formation of EVs vary: (**A)** Exosomes: These vesicles, ranging from 30 to 100 nm in diameter, originate from the endocytic pathway. Multivesicular bodies (MVBs), which form from early endosomes, have two potential fates. They can fuse with lysosomes for degradation, or merge with the plasma membrane, releasing exosomes into the extracellular space. (**B)** Microvesicles: These are larger, with diameters between 100 and 1000 nm, and form directly through the outward budding and shedding of the plasma membrane. The figure also illustrates a typical EV cargo system, encompassing diverse components such as proteins (e.g., CD9, CD63, CD81), lipids, nucleic acids, and non-coding RNAs. (**C)** Apoptotic bodies: These particles, typically ranging in diameter from 1 to 5 micrometers, are produced during the final stages of apoptosis through a complex process involving caspase activation, cellular morphological changes, and blebbing, which culminates in their engulfment and digestion by phagocytes to prevent inflammation and ensure tissue homeostasis.The endoplasmic reticulum (ER) is indicated for context

Weight loss and muscle wasting, whether caused by cancer cachexia or chemotherapy-induced cachexia, reduce patients’ tolerance to anti-cancer therapy and increase the risk of post-operative complications [13–15], and enervated cardiac and diaphragm muscles can normally be prone to earlier deaths from heart and lung failures [5, 16]. Furthermore, Patients with cancer associated cachexia often suffer from irreversible fatigue, decreased food intake and mobility, leading to reduced physical and emotional activity, impaired daily activity abilities and consequently a reduced quality of life [17, 18]. The complex interplay of tumor-derived factors and host responses orchestrates the development and progression of cachexia [19]. Effective management of cachexia requires early identification and a comprehensive approach, including nutritional support, targeted pharmacotherapy, and tailored exercise interventions, to improve patient outcomes and quality of life. Recently, EVs have emerged as momentous mediators in intercellular communication and have captured considerable attention in cancer studies. These small, membrane-bound particles released by cancer cells can encapsule pro-inflammatory cytokines and bioactive molecules that promote systemic inflammation, metabolic reprogramming, and muscle wasting. They facilitate communication between cancer cells and the tumor microenvironment, exacerbating the cachexia syndrome. Understanding the mechanisms of EVs in cachexia could lead to novel therapeutic strategies for this debilitating condition (Fig. 1).

**Fig. 2:**
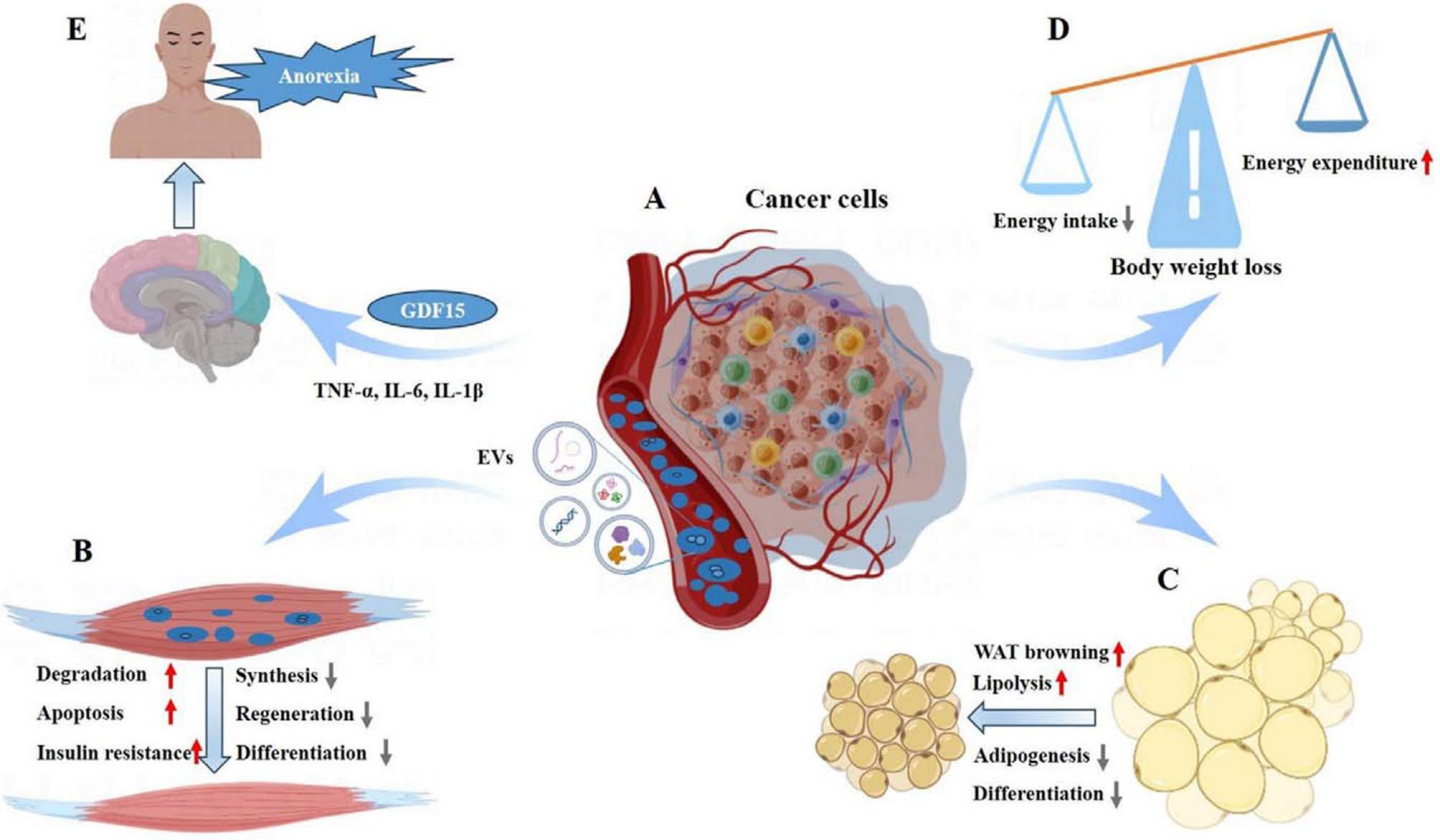
Impact of cancer cell-derived extracellular vesicles on recipient cells. This figure illustrates the role of EVs released by cancer cells in interacting with various recipient cells, affecting their functions and behaviors: (**A)** Intercellular Communication: EVs from cancer cells can travel to adjacent or distant cells. These EVs deliver a range of molecules, thereby modulating the behavior of recipient cells. (**B)** Muscle Cell Interaction: EVs originating from cancer cells carry specific cargoes that can trigger signaling pathways in muscle cells. This interaction influences muscle homeostasis and functionality. (**C)** Adipose Tissue Effects: Cancer-derived EVs can exert a direct or indirect impact on adipose tissue, contributing to adipose atrophy. (**D)** Metabolic Alterations: Tumor cell-derived EVs can transport bioactive molecules that affect metabolism, leading to decreased energy intake and increased energy expenditure, consequently causing body weight loss. (**E)** Appetite and Inflammation: Cancer cells may release EVs containing hormones, neurotransmitters, and pro-inflammatory factors. These contents can lead to diminished appetite or anorexia

As of now, three major subsets of EVs have been authenticated according to their diameter [20, 21]. Exosomes, the most well-studied subtype of EVs, participate in intercellular communication that are secreted by almost all kinds of cell types [22–24]. Enclosed in lipid bilayer structures, these EVs transport multifunctional biomolecules, with their bioactive cargo influencing every stage of human cancer development [25, 26], including cancer-induced cachexia **(**Fig. 2A**)**. They play a role both locally and systemically in regulating a wide array of physiological and pathological processes, particularly in cell-to-cell communication [27, 28]. Recent studies have shed light on the distinct roles of tumor-derived EVs compared to those derived from host tissues, particularly in the context of cancer progression and potential implications for cachexia development [29]. Cancer cell-derived EVs are implicated in various key aspects of tumor biology, such as promoting tumor progression, facilitating immune escape, and contributing to drug resistance [30]. They have been shown to alter metabolic processes, including glycolysis and lipid metabolism, through interactions with different cells within the tumor microenvironment. This includes influencing the behavior of cancer-associated fibroblasts and immune cells in ways that favor tumor growth and metastasis [31]. Furthermore, research tracking tumor cell-derived EVs in vivo has demonstrated that these EVs exhibit a specific distribution pattern and significantly alter the immune cell composition in target organs of metastasis [32]. Such findings emphasize the importance of tumor cell-derived EVs in cancer biology and offer insights into their potential role in cachexia development in cancer patients.

In addition to their role in the cachexia driven by EVs derived from tumors, EVs originated from host tissues, such as skeletal muscle, adipose tissue, and immune cells, have also been implicated in the pathogenesis of cancer-induced wasting. Studies have highlighted the contribution of muscle-derived EVs in promoting muscle wasting and systemic metabolic alterations observed in CC [33, 34]. These EVs have been shown to contain specific miRNAs, long non-coding RNAs, and proteins that can modulate crucial cellular processes, such as dynamic changes, and immune responses [35]. Furthermore, muscle-derived EVs can be taken up by neighboring cells, including adipocytes [36] and macrophages [37]. These interactions indicate a potential role in adipogenesis, influencing the metabolic profile and modulating inflammatory conditions that may be linked to cachexia.

Here, we will explore the emerging role of EVs in CC, highlighting their involvement in the intercellular communication network and their potential as diagnostic and prognostic markers, as well as therapeutic targets. Furthermore, we will discuss recent advancements in EV research, identify key challenges and opportunities in the field, and propose future directions for investigating the complex biology of EVs in the context of CC.

## Biogenesis, release, and uptake of EVs

EVs are diverse and can be categorized into several subsets based on their size and origin. Currently, three primary classes are recognized: exosomes (30–100 nm), microvesicles (100–1000 nm), and apoptotic bodies (1000–5000 nm) [38]. Exosomes, particularly well-researched, are formed through a unique process involving the budding of endosomal multivesicular bodies (MVBs) and the release of intra-luminal vesicles (ILVs) containing exosomes into the extracellular space [39]. This process contrasts with microvesicles, which are generated by the outward budding of the plasma membrane **(**Fig. 1A**)** [40]. On the contrary, apoptotic bodies form through the outward blebbing of the plasma membrane in cells undergoing apoptosis [41]. Before being released from parent cells, EVs encapsulate a wide variety of biological molecules, including proteins (e.g., TSG101, CD9, CD63, CD81, HSPs), lipids, nucleic acids, and non-coding RNAs **(**Fig. 1B**)**. The role of these EVs, especially exosomes [42] and microvesicles [43], is increasingly linked to the development of cachexia in cancer patients. During apoptosis, cells form apoptotic bodies containing cellular components, often leading to anti-inflammatory effects upon uptake by antigen-presenting cells or phagocytes **(**Fig. 1C**)** [44].

Different cell types in the tumor microenvironment, including cancer cells, stromal cells, and immune cells, secrete EVs [20, 45]. Their release is influenced by conditions common in cancer cachexia, such as hypoxia, cellular stress, oncogenic signaling, and cell proliferation, as well as interactions within the tumor microenvironment [45–50]. For instance, hypoxia can trigger endoplasmic reticulum (ER) stress, leading to the activation of the unfolded protein response (UPR). This response enhances EV secretion as a means of removing misfolded proteins from the cell [51]. It can also activate oncogenic signaling pathways, including PI3K/Akt, MAPK, and mTOR, known to regulate EV secretion in cancer cells [51, 52]. EVs harbor molecules that can enhance tumor progression, metastasis, and drug resistance, and modulate the behavior of adjacent cells and the immune response.

Once released, EVs can be taken up by recipient cells through mechanisms such as endocytosis, micropinocytosis [53], and direct fusion with the plasma membrane [54]. The uptake is influenced by the EVs’ cargo composition, surface proteins, and the type of recipient cell. In CC, EVs from cancer cells can be absorbed by different cell types, including skeletal muscle cells, adipocytes, and immune cells, delivering bioactive molecules that affect their function and phenotype. For example, cancer cell-derived EVs can induce muscle wasting by affecting muscle physiology, including myofibrillar protein degradation [55, 56]. and mitochondrial function in muscle cells [57]. Another study underscores the importance of the cargo carried by different types of tumor-derived EVs, as they can mediate various pathways of internalization into recipient cells and induce similar phenotypes through different mechanisms [58].They relatively contribute to adipose tissue dysfunction, such as lipolysis [58] and adipocyte atrophy [59], and can reach organs like the liver to induce metabolic changes [60] and inflammation [61], exacerbating cachexia. Overall, while it’s clear that EVs play a role in cancer progression and potentially in the development of cachexia, the specific differences in EVs between cachectic and non-cachectic cancers remain an area for further investigation.

## EVs and pathogenesis of CC

Although research on EVs in relation to CC is still in its early stages, there has been a significant enhancement in the depth and breadth of related studies. These studies offer new insights into the potential mechanisms driving the onset and progression of cancer-associated cachexia. The impact of tumor-derived EVs and their contents on cancer progression and the development of CC has become a focal point of considerable scientific interest and concern. Pitzer and colleagues have extensively reviewed the direct effects of tumor-derived exosomes on skeletal muscle and adipose tissue, as well as their intercellular communication, which are principal factors in the weight loss associated with cancer cachexia [62]. In the following section, we will delve into the interactions between EVs and cancer-induced cachexia, focusing on skeletal muscle wasting, loss of adipose tissue, systemic inflammation, metabolic alterations, and the regulation of central nervous system (CNS) homeostasis. Our aim is to provide a more comprehensive overview of the intricate interplay among these factors. Please refer to the specific role by which EVs contribute to cachexia in Fig. 2.

### EVs and skeletal muscle wasting

Muscle wasting and atrophy are significant characteristics of CC [63, 64]. Recent evidence indicates that EVs play a crucial role in the muscle wasting associated with CC [65]. Research has demonstrated that EVs originating from cancer cells directly influence skeletal muscle cells, leading to muscle wasting (Fig. 2A, B) [55, 56]. Furthermore, research has shown that EVs derived from cancer cells contain specific molecular cargo that can impact various cellular processes, especially those relevant to muscle tissue.

Cancer cell-derived EVs may contribute to muscle wasting through the transfer of specific molecules, such as non-coding RNAs. Notably, several studies have identified particular microRNAs (miRNAs) in EVs from cancer cells that are closely linked to muscle wasting [42, 43, 66–70]. Mechanistically, miRNAs transferred to skeletal muscle cells via EVs play a pivotal role in regulating gene expression linked to muscle protein synthesis and degradation. They influence key pathways, including TLR7 signaling [43, 66], Forkhead Box O (FoxO) Transcription Factors [70], the ubiquitin-proteasome system, and autophagy-lysosome pathways [71]. Additionally, EV-associated miRNAs impact essential signaling routes critical for muscle growth and atrophy, notably the insulin-like growth factor-1 (IGF-1) and mTOR pathways [72], which are vital for muscle protein synthesis. The specific roles and mechanisms of EV-encapsulated microRNAs in these processes are detailed in Table 1, highlighting their integral part in muscular function and health. Additionally, recent research has explored the role of EV-based circular RNAs (circRNAs) derived from tumor cells in contributing to muscle wasting [73].

**Table 1:** EV-encapsulated miRNAs from cancer cell in muscle wasting

Cancer type	Potential source	EV cargos	Targets	Functions	References
Colon cancer	C26	miR-195a-5p ↑ miR-125b-1-3p ↑	Bcl-2-apoptotic signaling.	Muscle atrophy ↑	[42]
Lung cancer Pancreatic cancer	LLC, A549 PC1, Panc-2, Miapaca-2	miR-21 ↑	TLR7	Myoblast apoptosis ↑	[43]
Lung cancer Pancreatic cancer	LLC AsPC1	miR-21 ↑ miR-29a ↑	TLR7	Myoblast apoptosis ↑ and cell death ↑	[66]
Colon cancer	C26	miR-183-5p ↑	Smad3	Protein degradation ↑ Mitochondrial respiration ↓	[67]
Oral squamous cell carcinoma	SCC7	miR-181a-3p ↑	ERS	Muscle atrophy and apoptosis ↑	[68]
Breast cancer	MCF-7	miR-122-5p ↑	TP53	Mitochondrial homeostasis ↓ Function of skeletal muscle ↓	[69]
Pancreatic cancer	PDAC	Nine miRNAs ↑	PI3K/Akt/FoxO1	Insulin resistance of skeletal muscle cells ↑	[70]

In addition to non-coding RNAs, EVs from cancer cells can induce pro-inflammatory responses and activate inflammatory pathways. Two key pro-inflammatory cytokines that can be upregulated are tumor necrosis factor-alpha (TNF-α) and interleukin-6 (IL-6) [74]. The activation of the NF-kB (Nuclear Factor-kappa B) pathway, a key event in this process, leads to the upregulation of pro-inflammatory genes [75]. Additionally, the IL-6-mediated STAT3 (signal transducer and activator of transcription 3) pathway is implicated in muscle inflammation and wasting [75, 76]. These EVs also impact other cell types within the muscle microenvironment, such as fibroblasts and adipocytes, leading to tissue remodeling and metabolic changes that further contribute to muscle wasting [77]. Moreover, cancer cell-derived EVs interfere with muscle regeneration by affecting the activation and differentiation of muscle cells [57]. These EVs can transport bioactive molecules like oxidized proteins, which, when internalized by muscle cells, disrupt the cellular redox balance, leading to oxidative stress and the degradation of contractile proteins [47, 78]. Furthermore, cancer cell-derived EVs may influence the contractile properties of muscle cells. These vesicles can carry factors that modify the expression and activity of proteins crucial for muscle contraction and force generation [58, 79, 80]. Collectively, these studies shed light on the molecular mechanisms by which tumor cell-released EVs mediate muscle wasting and affect muscle function in distally located muscles.

### EVs and adipose atrophy

Cancer cachexia frequently encompasses not only muscle wasting but also the loss of adipose tissue [80]. EVs released by cancer cells can have a direct or indirect impact on adipose tissue metabolism, accelerating the wasting of adipose tissue **(**Fig. 2C). Additionally, EVs secreted by cancer cells may carry pro-inflammatory factors that disrupt the normal functioning of adipose tissue, contributing to its degradation [59, 76]. This process can result in adipocyte atrophy and a significant loss of fat tissue, which is a hallmark of cancer cachexia.

Tumor-derived EVs may contribute to adipose wasting through the transfer of non-coding RNAs (ncRNAs), particularly microRNAs (miRNAs). Research has shown that tumor EVs can deliver specific miRNAs to adipose tissue, impacting various functions such as adipocyte differentiation, the conversion of white adipose tissues (WATs) to a more metabolically active state, lipolysis, lipid metabolism, and insulin sensitivity (Table 2) [81–87]. In addition to ncRNAs, tumor EVs may also carry proteins that directly influence adipose tissue biology. For instance, EVs from certain tumor types can contain factors that promote lipolysis, the process of breaking down stored fats [88, 89]. This activity can lead to an increased release of fatty acids from adipose tissue, contributing to its wasting.

**Table 2:** The roles of EV-enclosed miRNAs from cancer cell in adipose atrophy

Cancer type	Potential source	EV cargos	Targets	Functions	References
Lung cancer	A549 H1299 AGS	miR-425-3p ↑	PDE4B cAMP/PKA	Preadipocyte proliferation and differentiation ↓ Adipocyte lipolysis and WAT browning ↑	[81]
Colorectal cancer	HCT-116 HEK293T Patient sample	miR-146b-5p ↑	HOXC10	Lipolysis and WAT browning ↑	[82]
Chronic myeloid leukemia	K562	miR-92a-3p ↑	C/EBPα	Adipogenesis ↓	[83]
Breast cancer Gastric cancer	MCF-7 SGC7901 MGC803	miR-155 ↑	UBQLN1 C/EPBβ	Adipose loss ↑ Adipogenesis ↓ Brown adipose differentiation ↑	[84, 85]
Gastric cancer	Serum EV	miR-410-3p ↑	IRS-1	Adipogenesis, lipidosis and differentiation ↓	[86]
Breast cancer	BC cell lines (MDMB231, SK-BR-3, 4T1, E0771)	miR-204-5p ↑	Leptin signaling	Lipolysis and browning ↑	[87]

### EVs and systemic inflammation

**Fig. 3:**
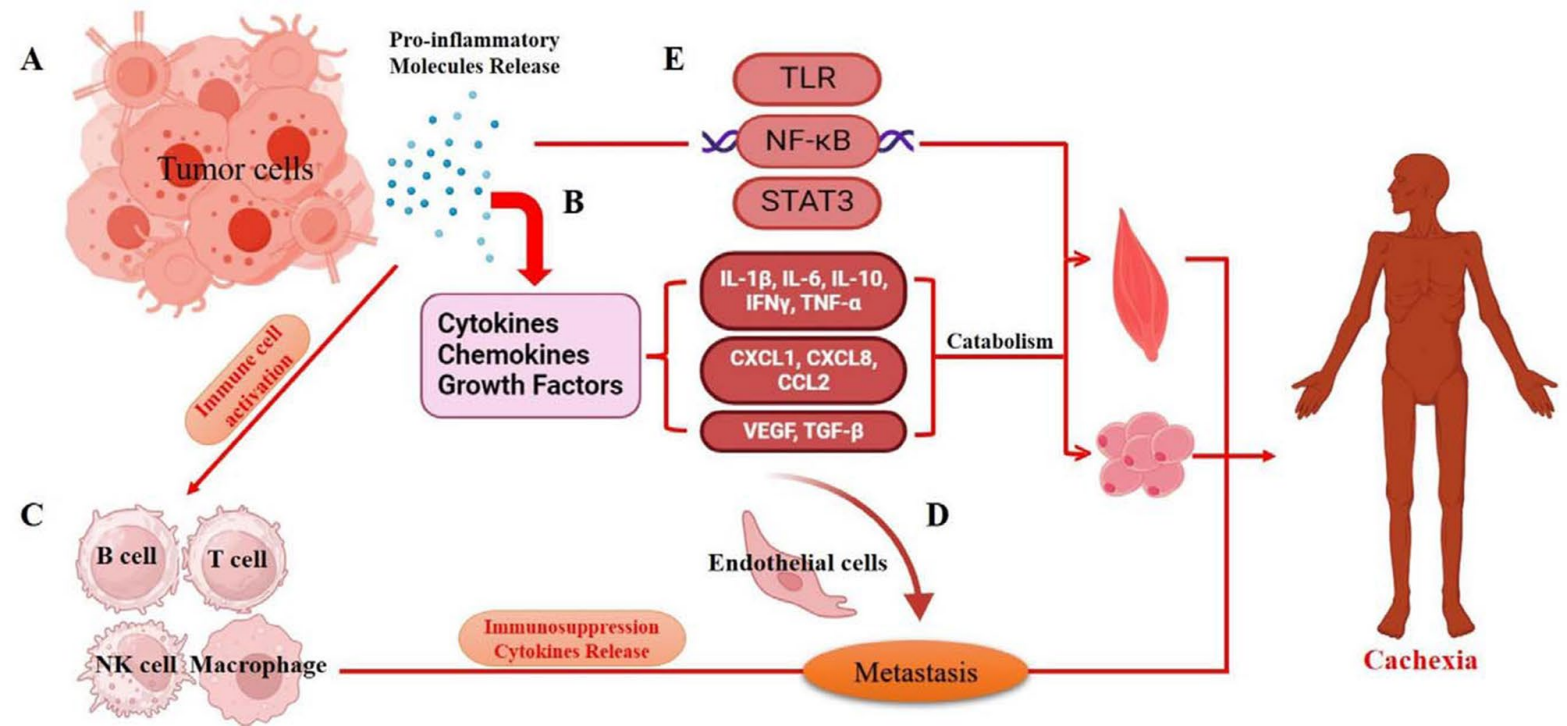
The role of inflammatory factors from tumor and immune cells in cancer cachexia. **(A)** EVs from tumor cells carry various pro-inflammatory molecules. (**B)** The molecules contained within EVs, such as cytokines, chemokines, and growth factors, contribute to cancer progression and accelerate the onset of cancer-associated cachexia. (**C)** Regulating inflammatory molecules leads to immunosuppression and stimulates cytokine release, which in turn boosts metastasis and speeds up cachexia. (**D)** EVs interact with endothelial cells to encourage angiogenesis and with lymphatic endothelial cells to aid in cancer dissemination. (**E)** Tumor-derived EVs are pivotal in triggering inflammation-related signaling pathways, specifically NF-κB, STAT3, and TLR pathways. *Abbreviations* NF-κB, Nuclear Factor-kappa B; STAT3, signal transducer and activator of transcription 3; TLR, toll-like receptor

EVs have been recognized as significant mediators of inflammation in various diseases, including cancer [90]. In CC, EVs released by either tumor cells or immune cells play a role in exacerbating inflammatory processes. Research has linked EVs to the promotion of tumor-associated inflammation, muscle wasting, and systemic metabolic imbalances in CC [76, 91]. EVs originating from tumor cells are known to carry a range of pro-inflammatory molecules (Fig. 3A), including cytokines such as IL-6, interleukin-1 beta (IL-1β), Interleukin-10 (IL-10), interferon-γ (IFNγ), and Tumor Necrosis Factor-alpha (TNF-α) [92]; chemokines like CXCL1, CXCL8, and CCL2 [93], and growth factors such as Vascular Endothelial Growth Factor (VEGF) and Transforming Growth Factor-beta (TGF-β) [94]. These molecules not only promote cancer progression but also accelerate the onset and development of cancer-related cachexia **(**Fig. 3B**)** [95–98].

Recent research has also illuminated the crucial immune cell pathways involved in weight loss and tissue degradation in patients with cancer cachexia [5, 99, 100]. Tumor-derived factors interact with a variety of immune cells and endothelial cells, significantly influencing the tumor microenvironment [100, 101]. These factors are normally packaged within EVs [102–104] and modulate the function of immune cells such as T cells [105], macrophages [106], and natural killer (NK) cells [107]. This regulation results in immunosuppression and stimulates the release of cytokines **(**Fig. 3C) [104]. Furthermore, these EVs interplay with endothelial cells, including vascular endothelial cells [108], to promote angiogenesis and with lymphatic endothelial cells to facilitate cancer spread through the lymphatic system. This complex interaction creates a tumor microenvironment that is immunosuppressive, promotes angiogenesis, and enhances metastasis, thereby accelerating cachexia **(**Fig. 3D). Additionally, these EVs can transport tumor-derived nucleic acids, like microRNAs, influencing the inflammatory response in recipient cells and delivering pro-inflammatory factors that reinforce the overall inflammatory state [109, 110]. . This multifaceted role of EVs in promoting inflammation underscores their significance in the pathophysiology of cancer cachexia and highlights their potential as targets for therapeutic intervention.

In the cachectic state, tumor-derived EVs are also critical in activating inflammatory-related signaling pathways, notably NF-κB [111, 112], STAT3 [113, 114], and toll-like receptor (TLR) signaling **(**Fig. 3E**)** [115, 116]. A study has highlighted that EVs from LLC (Lewis Lung Carcinoma) and C26 tumor cells can induce adipocyte wasting, an effect attributed to the action of interleukin-8 (IL-8). IL-8, present outside the adipocytes, activates the NF-κB signaling pathway [59]. Furthermore, extracellular vesicles from LLC tumor cells affect muscle cells by inducing atrophy and stimulating the breakdown of fat in adipocytes. These effects are mediated by the extracellular presence of IL-6, which triggers the STAT3 pathway within the target cells [76]. The interplay between inflammation and signaling pathways is pivotal in the development and progression of cachexia. Managing this condition often involves targeting these inflammatory processes. This is typically done through interventions such as anti-inflammatory medications or treatments aimed at modulating the involved signaling pathways, underscoring the importance of understanding these mechanisms for effective therapeutic strategies.

### EVs and metabolic alterations

Studies have shown that EVs from cancer cells or the tumor microenvironment frequently contain cargo molecules linked to metabolic disorders **(**Fig. 2D**)** [117, 118]. These molecules can be transferred to various recipient cells, including muscle cells, leading to alterations in their metabolic functions. One notable impact of cancer cell-derived EVs is the induction of insulin resistance in muscle cells, impairing their ability to uptake and utilize glucose [70]. This disruption in glucose metabolism is a key aspect of the metabolic reprogramming associated with cancer cachexia. In addition to affecting muscle cells, EVs can also influence lipid metabolism in adipocytes. They promote lipolysis, contributing to the systemic lipid imbalances commonly seen in CC [58, 59, 76, 81, 82, 85, 86, 119]. This alteration in lipid metabolism further exacerbates the wasting and systemic metabolic dysregulation characteristic of the condition. Moreover, EVs discharged by activated immune cells may transport inflammatory cytokines, contributing to the widespread systemic inflammation and metabolic dysregulation in CC [77].

Mitochondria, the cell’s energy-producing organelles, are critically linked to the development and progression of cancer cachexia [120]. EVs released by cancer cells can harbor specific cargo that directly affects mitochondrial function in recipient cells [57]. For instance, it has been demonstrated that tumor-derived EVs can induce mitochondrial dysfunction in skeletal muscle cells [69]. They also induce insulin resistance in muscle cells, leading to lipid accumulation and impairing glucose uptake and utilization [70]. A notable study uncovers the presence of mitochondria within EVs, suggesting a significant role in intercellular communication and tissue homeostasis [121]. The idea that EVs can transport entire mitochondria or mitochondrial components opens new avenues for understanding how cancer cells can influence the metabolism and function of distant tissues.

### EVs and CNS homeostasis regulation

EVs can significantly impact CNS homeostasis in CC, primarily through the transfer of pro-inflammatory molecules. EVs originating from tumor cells often contain pro-inflammatory cytokines and chemokines, including TNF-α, IL-6, and IL-1β **(**Fig. 2E) [122]. These EVs, when they interact with CNS cells, can trigger an inflammatory response, leading to the activation of glial cells, the release of additional pro-inflammatory mediators, and a disruption of CNS homeostasis [123]. This inflammatory response in the CNS, instigated by EVs, affects several processes that are crucial in CC. For instance, the pro-inflammatory cytokines released by EVs can influence hypothalamic nuclei responsible for appetite regulation, potentially leading to reduced food intake and anorexia [122, 124]. Moreover, TNF-α from EVs can promote muscle protein breakdown by activating pathways that lead to the degradation of muscle proteins, especially myofibrillar proteins [125]. It is important to recognize that the regulation of muscle atrophy by the CNS is a multifaceted process, influenced by an array of factors. The involvement of EVs and their pro-inflammatory cargo provides a deeper understanding of the complex interactions at play in the CNS during the progression of cancer cachexia.

In feeding regulation, particularly in CC, the role of EVs is increasingly recognized. Elevated levels of growth differentiation factor 15 (GDF15) are often observed in CC, and the presence of GDF15 in exosomes may be a contributing factor to the appetite suppression and weight loss experienced by cancer patients **(**Fig. 2E) [126, 127]. GDF15 is known for its role in mediating anorexic responses, and its presence in EVs suggests a pathway by which tumors can systemically affect appetite and energy balance. Additionally, EVs can influence the sympathetic nervous system’s activity [128], leading to increased energy expenditure [129] and contributing to muscle wasting [130]. This interaction further exemplifies the complex ways in which EVs can modulate systemic metabolic responses in cancer cachexia. Beyond their effects on metabolism and appetite, EVs derived from tumor cells also carry molecules that can promote tumor growth and metastasis [45, 131]. These EVs can facilitate the spread of cancer cells to distant sites, including the CNS. Once in the CNS, cancer cells and their EVs can disrupt normal cellular functions and promote inflammation. This can exacerbate the progression of cachexia, highlighting the multifaceted impact of EVs in the pathophysiology of cancer and its systemic effects.

Additionally, research into small extracellular vesicles (sEVs) and their regulatory effects on hypothalamic AMPK (AMP-activated protein kinase) function is a case in point. Studies have shown that peripheral intravenous administration of specific sEVs can directly target neuronal cell populations in the hypothalamus [132, 133]. This targeted approach holds potential for treating cancer cachexia, particularly considering the interplay between hypothalamic AMPK activity, elevated brown adipose tissue (BAT) thermogenesis, and the browning of white fat [134–136]. Furthermore, characterizing EVs present in biofluids like blood or cerebrospinal fluid could provide valuable diagnostic and prognostic insights for cancer cachexia. By analyzing these biofluids for specific EVs and their cargo, clinicians may gain a better understanding of the disease’s progression and the effectiveness of treatments.

### EVs as biomarkers of CC

Early detection and monitoring of CC are essential for effective management and treatment [137, 138]. Research on CC biomarkers has largely focused on mediators of skeletal muscle loss, produced by both tumor and host tissues. These include cachexia-inducible factors [139], pro-inflammatory cytokines [140, 141], lipids [142, 143], metabolic products of protein and fat [144], and non-coding RNAs [145–147]. However, none of these biomarkers have been extensively used in clinical practice to detect skeletal muscle wasting. Notable reviews by Loumaye et al. [148] and Cao et al. [149] discuss various CC biomarkers, emphasizing the identification of these markers and measuring their circulating levels in certain cancer types. In CC, the body undergoes significant metabolic and systemic changes, and both tumor and host tissues contribute to the altered profile of circulating EVs. It appears that the specific literature focusing on the relative contributions of tumor-derived versus host tissue-derived EVs in cancer cachexia, especially in the context of their presence in the circulation, is not readily accessible or may not be extensively covered in available research. This paper highlights recent developments in EVs as potential CC biomarkers, offering a foundation for future clinical research in this area.

### EVs incorporating proteins as biomarkers

EVs can transport proteins that reflect cachexia-related processes, including systemic inflammation, muscle wasting, and metabolic changes. Analyzing the proteomic content of EVs can pinpoint specific proteins or patterns related to CC. This approach holds promise for creating diagnostic and prognostic tools for CC.

### Inflammatory and metabolic markers

EVs carry numerous inflammatory cytokines from both host tissues and tumors, a process extensively studied in various cachexia models [138, 150]. IL-6 emerges as one of the most promising biomarkers for CC. IL-6 levels are directly associated with tumor stage, weight loss, and survival in lung and gastrointestinal cancer patients, as evidenced by studies [151, 152]. Additionally, potential biomarkers like TNF-alpha, β-dystroglycan, Monocyte Chemoattractant Protein-1 (MCP-1), IL-1β, and IL-8, originating from tumors and/or host tissues, show significant potential in CC diagnostics [140, 153, 154]. These bioactive molecules are integral to the inflammatory and metabolic alterations seen in cancer cachexia. Their levels in the body can provide insights into the severity of cachexia, and they offer potential targets for therapeutic interventions aimed at mitigating the devastating effects of this condition.

### Non-inflammatory markers

Numerous studies have highlighted specific proteins carried by EVs that are associated with CC. These proteins impact target cells and contribute to cachexia development. Notably, EV-Growth and GDF15 from cancer cells emerge as key molecules in CC research [126, 155, 156]. Another significant protein, the Proteolysis-Inducing Factor (PIF), secreted by tumor cells, is known to cause muscle wasting [156, 157]. It can be transferred via exosomes, leading to muscle protein degradation. Additionally, exosomal Fatty Acid Binding Protein 4 (FABP4) has been implicated in muscle wasting and systemic inflammation in CC [158–160]. Each of these proteins contributes to the multifaceted nature of cachexia through different mechanisms. These proteins’ interactions and effects underscore the complexity of cachexia, making it a challenging condition to manage and treat effectively. Further exploration in this field could yield valuable markers for CC prevention and treatment.

### Heat shock proteins (HSPs) cargo

Recent research has also highlighted a notable link between EVs and heat shock proteins (HSPs) in CC [55, 161]. It’s been discovered that certain HSPs are enclosed within EVs and released into the extracellular environment [162]. Furthermore, changes in HSP expression and activity have been noted in CC [163–165], indicating their role in cachexia’s development and progression. For example, increased levels of HSP70/90 are found in cachectic cancer patients compared to non-cachectic ones [166–169]. Additionally, higher EV concentrations in the serum of cancer patients have been associated with poorer prognosis [170, 171]. HSP27, another heat shock protein, has also been studied in CC contexts, with heightened levels detected in the skeletal muscles of cachectic cancer patients [172]. Another important study suggests that, in muscle-related diseases, upregulation of HSP70 and HSP90 may occur as a cellular response to alleviate protein folding stress and maintain protein homeostasis [164], although it’s essential to emphasize that this result is not specifically grounded in the context of CC. These insights underscore the potential of EV-associated HSPs as crucial molecular markers in CC management.

### Skeletal muscle biomarkers

Research has pinpointed muscle-specific proteins as promising biomarkers linked to muscle wasting in CC [173, 174]. Myostatin, known for inhibiting muscle growth, is notably elevated in EVs from cachectic cancer patients [175]. Additionally, proteins crucial for muscle protein synthesis and degradation, such as Bone Morphogenetic Proteins (BMPs), irisin, TGFβ, activin A, atrogin-1, and Muscle RING-Finger Protein-1 (MuRF1) [176, 177], have been identified as potential EV-derived biomarkers. These protein changes are closely linked to the progression and severity of the disease, underscoring their value in diagnosis and prognosis. Regular tracking of these EV-related muscle-specific proteins could offer significant insights into the effectiveness of treatments and the progression of CC over time.

## EVs incorporating miRNAs and RNAs as biomarkers

MicroRNAs (miRNAs), long non-coding RNAs (lncRNAs), and circular RNAs (circRNAs) are crucial regulators of gene expression and cellular processes, each playing a distinct yet interconnected role [178]. These RNA types are intricately involved in the complex cellular dynamics of cancer cachexia, influencing muscle metabolism, inflammation, and metabolic changes associated with this condition. Their collective dysregulation in cancer cachexia underscores their importance in the pathophysiology of this syndrome, presenting potential targets for therapeutic intervention. RNA molecules transported by EVs offer critical insights into the biological mechanisms underlying CC. Notably, specific types of RNA, including miRNAs, lncRNAs, and circRNAs, are often dysregulated in EVs from CC patients [179]. These altered RNA profiles mirror the molecular shifts tied to muscle wasting and the metabolic changes characteristic of cachexia.

## Cancer cell derived EVs -miRNAs and RNAs as biomarkers

Research has revealed specific miRNAs that are dysregulated in EVs derived from CC patients compared to non-cachectic individuals [180]. Various miRNAs, including miR-195a-5p, miR-125b-1-3p [42], miR-21, miR-29a [43, 66], miR-181a-3p [68], and miR-122-5p [69], are linked to muscle wasting and inflammation in CC. Additionally, miR-486 has been identified as a potential marker for cachexia severity and treatment response [181]. These miRNAs, often associated with cancer progression and muscle atrophy, are notably stable in the circulatory system when carried by EVs like exosomes. This stability positions them as effective biomarkers for cancer-related cachexia.

Beyond miRNAs, the potential of lncRNAs and circRNAs incorporated in EVs as biomarkers for CC is an area of active research, though their clinical applicability requires further validation. Initial studies have yielded promising results. For instance, lncRNAs like H19 [182] and LINC00355 [183] show different expression levels in EVs from cancer patients at risk of cachexia compared to healthy individuals, suggesting their utility in diagnosing or predicting CC. Other lncRNAs, such as Metastasis Associated Lung Adenocarcinoma Transcript 1 (MALAT1) [184] and HOX transcript antisense intergenic RNA (HOTAIR) [185], have been linked to CC and are detectable in EVs [185, 186]. Regarding circRNAs, current research on cachexia is limited, but there are studies like one on CircPTK2 that illustrate its role in lipid metabolism regulation in CC [187]. The inherent stability of EV-incorporated circRNAs, due to their resistance to exonuclease degradation, makes them promising candidates for biomarker exploration.

### Muscle specific EVs -miRNAs as biomarkers

The expression profiles of muscle-secreted miRNAs have been extensively studied, revealing their significant role in regulating muscle metabolism during CC [33, 145, 188]. Muscle-specific miRNAs, including miR-1, miR-133a, miR-133b, miR-206, miR-208a, miR-208b, and miR-499, along with muscle-enriched miR-486, are known to influence myogenesis, proliferation, differentiation, apoptosis of myotubes, and protein synthesis in skeletal muscle of CC patients [189, 190]. Recent research demonstrates that exosomes from skeletal muscle, containing myomiRs such as miR-1, miR-133a, miR-133b, and miR-206, play a dual role: they enter the circulatory system and facilitate inter-tissue communication between muscles, offering insights into new therapeutic approaches for muscle function [190]. However, detecting skeletal muscle-specific EVs remains challenging due to the complexity of EV types and a lack of specific markers for skeletal muscle-derived EVs. Nonetheless, these circulating EVs with muscle miRNAs, which contribute to muscle wasting, may serve as accessible and promising biomarkers in CC management.

### EVs-based lipid composition as biomarkers

Lipidomic analysis of EVs could uncover lipid signatures serving as diagnostic markers for cachexia. For example, Fan et al. [191] demonstrates that lipid profiles of plasma exosomes can distinguish early-stage lung cancer from healthy individuals. Similar studies have utilized serum or plasma exosomal lipids in diagnosing pancreatic cancer [192], breast cancer [193], and colorectal cancer [194–196], suggesting the potential of lipid biomarkers in EVs for diagnosing various cancer stages. While cancer and its treatments can significantly impact the body’s metabolism and energy balance, the specific effects on adipose tissue are not well-documented [197]. Therefore, significant research is still required in exploring EV-encapsulated lipids as potential biomarkers for cancer-related cachexia.

## EVs as therapeutic agents of CC

**Fig. 4:**
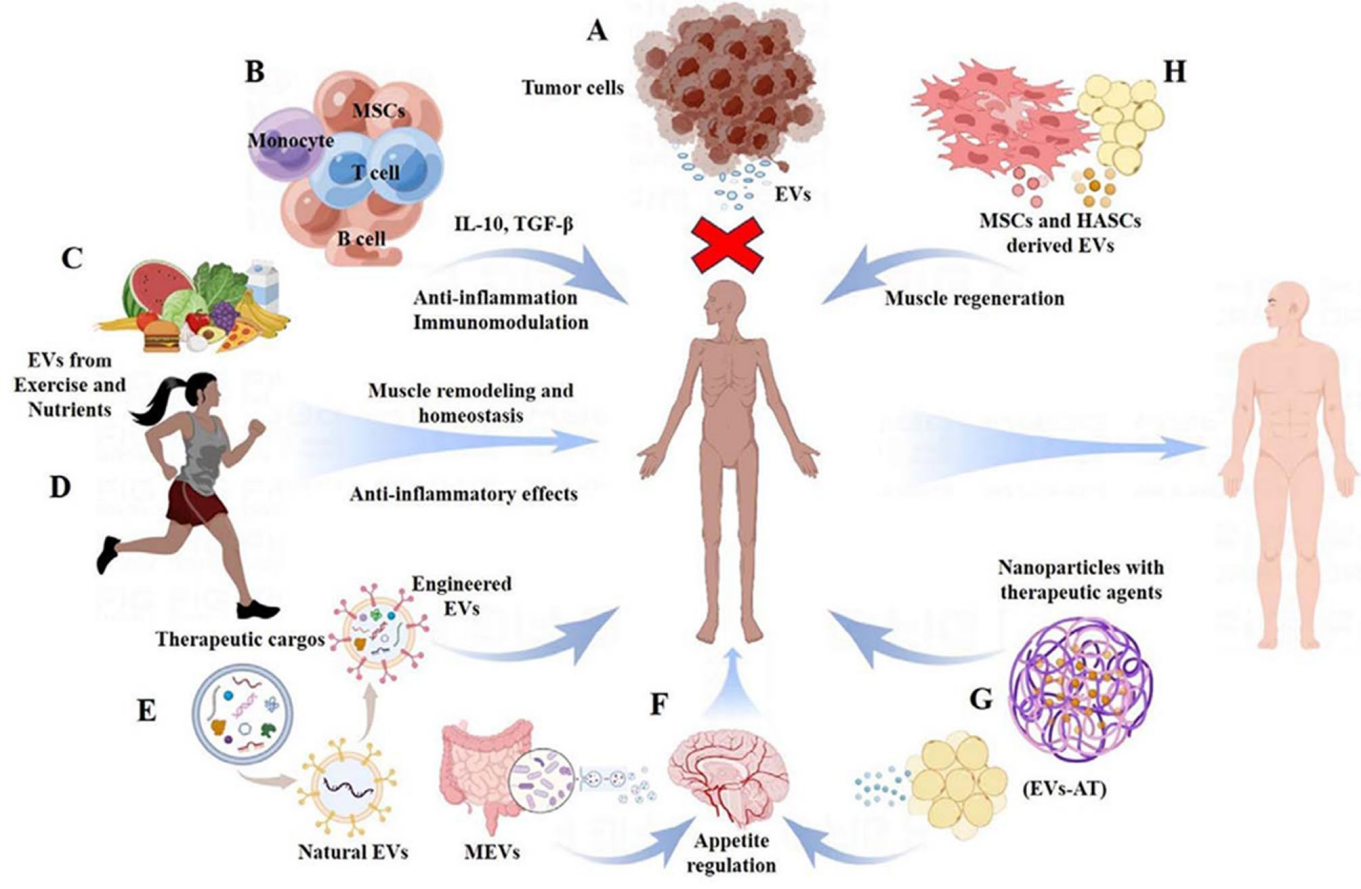
Therapeutic potential of EVs in cancer-induced cachexia. EVs have demonstrated promising therapeutic effects in managing cancer-induced cachexia, through various mechanisms: (**A)** Inhibition of EV Production: Reducing the production and release of cancer cell-secreted EVs can alleviate symptoms of cancer cachexia. (**B)** Immune Cell-Derived EVs: EVs from immune cells or anti-inflammatory sources carry factors that can suppress inflammation and modulate the immune response, thus helping to manage cancer cachexia. These EVs can regulate immune responses, diminish systemic inflammation, and restore immune homeostasis, potentially slowing the progression of cachexia. (**C)** Nutrient-Derived EVs: EVs carrying nutrients or those derived from nutrient sources are being explored for their therapeutic effects in cancer cachexia. (**D)** Exercise-Induced EVs: EVs generated through exercise may improve body weight, muscle mass, and physical performance in cachectic patients. (**E)** Cargo Modulation: Altering the cargo of EVs presents a novel approach for treating cancer cachexia. (**F)** Gut and Adipose Tissue-Derived EVs: These EVs could regulate appetite by affecting signaling pathways in the brain’s appetite-control centers. (**G)** Loaded EVs: EVs can be engineered to carry anti-inflammatory agents, specific anti-cancer drugs, and other therapeutic agents, making them effective for therapeutic cargo delivery. (**H)** MSC and HASC-Derived EVs: Mesenchymal stem cell (MSC) and human adipose-derived stem cell (HASC)-derived EVs have shown promise in enhancing muscle tissue regeneration in preclinical studies. *Abbreviations* MSC, mesenchymal stem cell; HASCs, human adipose-derived stem cells; EVs-AT, adipose tissue-derived EVs; MEVs, microbiota-derived extracellular vesicles

EVs indeed have gained attention in the field of medicine and therapeutics for their potential in treating various diseases, including cancer cachexia. These vesicles can originate from various cell types, such as mesenchymal stem cells, immune cells, and even tumor cells. Their ability to influence cellular processes and modulate immune responses makes them promising candidates for therapy. In this context, we focus specifically on the emerging role of EVs as therapeutic agents in CC, as detailed in Fig. 4.

### Inhibiting production and release of cachectic EVs

The hypothesis is that EVs released by cancer cells or other cells contribute to the systemic effects of cachexia by transferring factors that promote muscle wasting, appetite suppression, and metabolic changes. Additionally, tumor-derived EVs can exacerbate cachexia by sending pro-cachectic signals to distant tissues. Therapeutically, targeting these tumor-derived EVs [198–201] or blocking their pathway activation [43, 56, 67] can mitigate cachexia-related complications. For example, amiloride, a commonly used diuretic, significantly improves metabolic disorders in cachectic gastrocnemius by effectively inhibiting tumor-derived exosome release, thus affecting muscle catabolism, protein synthesis, glycolysis, and ketone body oxidation [201]. Similarly, GW4869, an inhibitor of exosome production and release, shows potential in reducing lipolysis and adipose tissue browning in cachexia [198]. Moreover, IMO-8503 is found to suppress cancer cells’ release of EVs containing circulating miRNAs, thereby reversing cachexia with minimal side effects [66]. Another study indicates that omeprazole, a proton pump inhibitor (PPI), can ameliorate cancer-induced cachexia by limiting the release of EV surface proteins like Hsp70 and Hsp90 [202]. Additionally, blocking CD81 on EVs from senescent bone marrow-derived mesenchymal stem cells (BMSCs) can reduce muscle wasting [203], suggesting its potential in preventing muscle loss. Thus, suppressing EV release emerges as a promising cell-free therapeutic approach for CC treatment **(**Fig. 4A).

### EVs as anti-inflammatory agents

Recent research indicates that EVs from certain cell types, like mesenchymal stem cells (MSCs) or immune cells, may have anti-inflammatory effects, suggesting their potential use as therapeutic agents in CC (Fig. 4B). These EVs can interact with and modulate immune cell functions. For instance, EVs from MSCs or immune cells [204–207] often carry anti-inflammatory proteins like IL-10 or TGF-β, which can suppress immune responses and reduce inflammation. Moreover, they can influence macrophage polarization, shifting them from a pro-inflammatory (M1) state to an anti-inflammatory (M2) state [208]. This shift towards M2 macrophages could lessen the inflammatory response, thereby potentially mitigating cachexia-related inflammation. Additionally, EVs transport specific miRNAs to target cells, impacting various signaling pathways involved in inflammation [209]. Certain miRNAs in EVs have demonstrated anti-inflammatory properties [210], offering a possible way to alleviate inflammation in CC.

### Exercise related EVs for treating CC

Physical exercise is a key strategy to counteract muscle atrophy and dysfunction in CC [211, 212]. During physical activity, EV trafficking plays a crucial role in inter-tissue communication [213–216]. Originating from muscle, immune, or cancer cells, these EVs can mediate the systemic effects of exercise on diverse tissues and organs [181]. , potentially improving cachexia symptoms.

Exercise-induced EVs are gaining attention for their roles in oncology [181] and skeletal muscle [217]. They are particularly interesting for their possible correlation with muscle remodeling and homeostasis [218]. The molecules within these EVs can help modulate processes like reducing oxidative damage, influencing mitochondrial function [219] improving metabolism [220] and enhancing skeletal muscle insulin sensitivity **(**Fig. 4D) [221]. Physical training can alter the number of circulating EVs and their protein contents. For example, exercise can increase the release of EVs containing Hsp72 [216] and Hsp60 [222], with Hsp60 activating the Peroxisome Proliferator-Activated Receptor Gamma Coactivator 1-Alpha (PGC1α) pathway, crucial for modulating muscle wasting [72, 222]. However, it’s important to note that these findings are not specifically studied in the context of CC. The precise mechanisms and therapeutic uses of exercise-induced EVs in CC remain areas of active research.

In addition to combatting muscle dysfunction, EVs from physical exercise may help delay CC progression through their anti-inflammatory effects **(**Fig. 4D). Exercise-induced muscle-derived IL-6 has shown to inhibit other inflammatory factors, exerting anti-inflammatory impacts [223]. EVs released post-exercise carrying meteorin-like protein can increase delivery of anti-inflammatory cytokines [224, 225]. The ability of contracting skeletal muscle cells to communicate with other organs via EV-based humoral factors is key to how physical exercise induces systemic adaptations, enhancing overall health [226]. These insights suggest a significant role for exercise-induced EVs in cachexia treatment.

### EVs as drug delivery system

Studies involving animal models and preclinical research have demonstrated the potential of EVs as a drug delivery system for muscle atrophy. For instance, EVs derived from MSCs have shown promise in promoting muscle regeneration, reducing inflammation, and improving muscle function in models of muscle atrophy **(**Fig. 4H**)** [227–230]. Additionally, research indicates that exosomes released from differentiating human skeletal myoblasts can stimulate myogenesis in human adipose-derived stem cells (HASCs), thereby accelerating muscle regeneration **(**Fig. 4H**)** [231]. Furthermore, the development of artificial nanovesicles, especially exosome-mimetic nanovesicles, is attracting interest in cancer research as potential therapeutic agents [232]. These nanovesicles are designed to replicate the properties of natural exosomes, potentially functioning as effectively or even more so [233]. Exosome-like systems, based on nanotechnology and surface engineering approaches, aim to overcome limitations of natural exosomes, showing potential as a competitive approach for innovative targeted anti-cancer therapies [234]. Nanoparticles, especially green nanoparticles with their environmental and biocompatibility benefits [235], are pivotal in cancer treatment as a drug delivery system [236–238]. Therefore, artificial nanovesicles and precision delivery systems may greatly improve treatment efficacy for cachexia in cancer **(**Fig. 4G).

### Modulating EV cargo as therapeutic agents

Modifying the content of EVs presents a promising therapeutic strategy for CC. Engineering EVs or altering their cargo-loading process could enable the delivery of specific molecules to mitigate the effects of cachexia **(**Fig. 4E). For instance, EVs could be loaded with anti-inflammatory agents [239], anchor factors [240], or myostatin inhibitors [241] to reduce muscle wasting. Additionally, engineering EVs to display specific targeting ligands on their surface would allow them to selectively bind to receptors on CC-related cells. An example of this is Physiactisome, a nanovesicle incorporating Hsp60, which mimics the beneficial effects of exercise training in combating muscle atrophy and cachexia [242]. Further, by manipulating the cargo of EVs, the adverse impacts of cancer-derived EVs on cachexia might be lessened. Research efforts could focus on selectively removing or altering specific proteins or nucleic acids within EVs linked to cachexia progression. Another approach involves loading therapeutic agents into EVs, turning them into targeted delivery vehicles for specific cells or tissues implicated in cachexia. Recent perspectives suggest that artificial EVs could be more effective than natural vesicles for drug delivery [243].

### EVs in ameliorating nutrient uptake and metabolism

EVs are known to transport a variety of bioactive factors capable of influencing the expression and activity of nutrient transporters. EVs from healthy cells, for example, may contain molecules that boost the uptake of essential nutrients like amino acids, glucose, and fatty acids by recipient cells. This enhancement in nutrient absorption can promote anabolism, helping to counteract the metabolic imbalances caused by CC **(**Fig. 4C**)** [244, 245]. On the other hand, CC is often accompanied by changes in gut microbiota composition, which can impact nutrient absorption and metabolism. EVs originating from specific bacterial strains, or those engineered to carry beneficial microbiota-derived molecules, might be capable of modifying the gut microbiota. Such alterations could potentially improve nutrient absorption and overall metabolism in CC patients [246].

### EVs in regulating appetite

Recent studies highlight the role of EVs from the hypothalamus and adipose tissue in regulating feeding behavior and energy balance [247–249]. Adipose tissue-derived EVs (EVs-AT) have been found to influence appetite-regulating pathways by altering the expression of appetite-related genes in target cells **(**Fig. 4F) [248]. Furthermore, EVs released by gut cells, including enteroendocrine cells, may contain bioactive molecules that facilitate signaling between the gut and the brain [250], thereby modulating appetite pathways and affecting satiety and hunger cues [251, 252]. Additionally, microbiota-derived extracellular vesicles (MEVs), released by gut bacteria, interact with host cells involved in appetite regulation. These MEVs can transport various bioactive molecules that influence appetite-related pathways **(**Fig. 4F**)** [253, 254]. However, it is important to note that more research is needed to fully understand the role of EVs in appetite regulation, particularly in the context of CC, and to investigate their potential as therapeutic targets.

### EVs as immunotherapeutic tactics

The use of EVs for immunomodulation in CC is a burgeoning area of research. EVs have the capability to influence the activity and function of various immune cells implicated in CC, such as MSCs, macrophages, B cells and T cells (Fig. 4B). Notably, they can shift macrophages from a pro-inflammatory M1 phenotype to an anti-inflammatory M2 phenotype [208]. This polarization shift can reduce tissue damage and facilitate tissue repair. Moreover, EVs might also boost the cytotoxicity of T cells against tumor cells [255, 256], aiding in tumor suppression and potentially easing the effects of cachexia. Therefore, manipulating the immune response via immune cell-derived EVs could significantly impact the progression of CC.

## Current perspectives and future challenges

EVs have emerged as significant players in the field of CC due to their vital role in intercellular communication and their potential in diagnostic and therapeutic applications. These vesicles, containing a diverse array of molecules such as proteins, nucleic acids, and lipids, mirror the phenotype of their originating cells, making them promising candidates for diagnostic and prognostic biomarkers in CC. Identifying specific EV biomarkers could enhance early detection and provide insights into cachexia’s progression. Furthermore, the ability to engineer EVs to deliver targeted treatments, such as small interfering RNAs (siRNAs) or drugs, directly to affected cells presents a novel avenue for CC therapy. However, the development of effective methods to load EVs with therapeutic agents and ensure their precise delivery to target tissues, such as muscle or adipose tissue, remains a challenge.

The potential of EVs in CC treatment is substantial, yet it is hindered by several technical obstacles. Standardizing methods for EV isolation and characterization, developing sensitive assays for cargo analysis, and scaling up therapeutic EV production are critical steps that need to be addressed [257–259]. Moreover, ensuring robust and reproducible methodologies is essential for the reliability and comparability of EV-based studies [260]. As EV-based approaches transition from preclinical to clinical settings, overcoming regulatory hurdles, establishing scalable manufacturing processes, and conducting comprehensive clinical trials to assess safety and efficacy become paramount [261]. Given the complexity of cancer cachexia, which varies among individuals and cancer types, and the diversity in EV cargo and composition, personalized approaches targeting specific cachexia mechanisms are necessary.

In total, while EVs offer significant promise in the management of cancer cachexia, overcoming the challenges associated with their development is crucial for realizing their full potential as diagnostic and therapeutic tools. A deeper understanding of EVs’ role in muscle wasting and metabolic dysfunction, coupled with ongoing research, collaboration, and technological innovation, is key to advancing the field. Integrating EV-based therapies with existing interventions, such as nutritional support, exercise, or pharmacological treatments, could provide synergistic effects, improving patient outcomes. Therefore, exploring multimodal approaches that combine EVs with other therapeutic modalities is essential for advancing the management of cancer cachexia.

## Conclusions

EVs are vital in cancer biology, mainly because they facilitate intercellular communication by transporting bioactive molecules. This function is critical for various cancer-related processes, including the development and spread of tumors, evasion of the immune response, and development of resistance to drugs. The composition of EVs, which mirrors the condition of their originating cells, offers significant insights for diagnosing, prognosticating, and tracking cancer progression. Moreover, their capacity to traverse biological barriers makes them promising for creating targeted drug delivery systems, potentially transforming cancer treatment strategies. While EVs hold great potential for cancer therapy, challenges such as achieving specificity to cancer cells, scaling up production, efficiently loading and securing the stability of therapeutic agents, and overcoming regulatory and safety obstacles, remain to be addressed.

EVs also play a crucial role in cancer-associated cachexia, a condition prevalent in advanced cancer stages that significantly affects patient quality of life and survival. By mediating interactions between tumor cells and the tumor microenvironment, EVs contribute to cachexia through inflammatory responses, metabolic reprogramming, and direct effects on muscle and adipose tissue. Understanding the role of EVs in cachexia is vital for early detection, monitoring, and identifying therapeutic targets to alleviate the condition’s impact on patient outcomes. Research has shown the diagnostic and prognostic potential of EVs in cancer cachexia, with ongoing studies exploring their therapeutic possibilities. The integration of EV-based biomarkers and therapies into clinical practice promises to improve patient outcomes by enabling earlier diagnosis, more accurate prognosis, and personalized treatment strategies. As knowledge of EV-mediated molecular mechanisms expands, targeted interventions can be developed, highlighting the importance of continued research and investment in this area. Overall, EVs represent a significant advancement in managing and treating cancer cachexia, offering a new frontier in cancer care.

## Data Availability

Not applicable.

## Abbreviations

CC: Cancer cachexia
EVs: Extracellular vesicles
MVBs: Endosomal multivesicular bodies
ILVs: Intra-luminal vesicles
CNS: Central nervous system
IGF-1: Lnsulin-like growth factor-1
mTOR: mechanistic target of rapamycin
IL-1β: L Interleukin-1 beta
IL-6: Interleukin-6
IL-10: Interleukin-10
IFN-γ: Interferon-γ
TNF-α: Tumor necrosis factor-alpha
VEGF: Vascular endothelial growth factor
TGF-β: Transforming growth factor-beta
NK: Natural killer
NF-κB: Nuclear factor-kappa B
STAT3: Signal transducer and activator of transcription 3
TLR: Toll-like receptor
LLC: Lewis lung carcinoma
GDF15: Growth differentiation factor 15
AMPK: AMP-activated protein kinase
BAT: Brown adipose tissue
MCP-1: Monocyte chemoattractant protein-1
PIF: Proteolysis-inducing factor
FABP4: Fatty acid binding protein 4
HSPs: Heat shock proteins
BMPs: Morphogenetic proteins
MuRF1: Muscle RING-finger protein-1
MALAT1: Metastasis ssociated lung adenocarcinoma transcript 1
HOTAIR: HOX transcript antisense intergenic RNA
BMSCs: Bone marrow-derived mesenchymal stem cells
MSCs: Mesenchymal stem cells
PGC1α: Peroxisome proliferator-activated receptor gamma coactivator 1-alpha
HASCs: Human adipose-derived stem cells
EVs-AT: Adipose tissue-derived EVs
MEVs: Microbiota-derived extracellular vesicles
